# Activated Kras^G12D^ is associated with invasion and metastasis of pancreatic cancer cells through inhibition of E-cadherin

**DOI:** 10.1038/bjc.2011.31

**Published:** 2011-03-01

**Authors:** S Rachagani, S Senapati, S Chakraborty, M P Ponnusamy, S Kumar, L M Smith, M Jain, S K Batra

**Affiliations:** 1 Department of Biochemistry and Molecular Biology, University of Nebraska Medical Center, Omaha, NE, USA; 2 Department of Biostatistics, University of Nebraska Medical Center, Omaha, NE, USA; 3 Eppley Institute for Research in Cancer and Allied Diseases, University of Nebraska Medical Center, Omaha, NE 68198-5870, USA

**Keywords:** activated Kras, invasion, metastasis, motility, pancreatic cancer

## Abstract

**Background:**

Pancreatic cancer (PC) harbours an activated point mutation (Kras^G12D^) in the Kras proto-oncogene that has been demonstrated to promote the development of PC.

**Methods:**

This study was designed to investigate the effect of the oncogenic Kras^G12D^ allele on aggressiveness and metastatic potential of PC cells. We silenced the oncogenic Kras^G12D^ allele expression in CD18/HPAF and ASPC1 cell lines by stable expression of shRNA specific to the Kras^G12D^allele.

**Results:**

The Kras^G12D^ knockdown cells exhibited a significant decrease in motility (*P*<0.0001), invasion (*P*<0.0001), anchorage-dependent (*P*<0.0001) and anchorage-independent growth (*P*<0.0001), proliferation (*P*<0.005) and an increase in cell doubling time (*P*<0.005) *in vitro* and a decrease in the incidence of metastases upon orthotopic implantation into nude mice. The knockdown of the Kras^G12D^ allele led to a significant increase in the expression of E-cadherin (mRNA and protein) both *in vitro* and *in vivo*. This was associated with a decrease in the expression of phoshpo-ERK-1/2, NF-*κ*B and MMP-9, and transcription factors such as *δ*EF1, Snail and ETV4. Furthermore, the expression of several proteins involved in cell survival, invasion and metastasis was decreased in the Kras^G12D^ knockdown cells.

**Conclusions:**

The results of this study suggest that the Kras^G12D^ allele promotes metastasis in PC cells partly through the downregulation of E-cadherin.

Pancreatic cancer (PC) has one of the worst prognoses among all known cancers, with a mortality to incidence ratio of ∼0.83 (Jemal *et al*, 2009). In the United States, it remains the fourth leading cause of cancer-related deaths with an incidence of ∼12.3 per 100 000 people (Jemal *et al*, 2009). The median survival of patients with PC is a mere 4.1 months with the overall 5-year survival rate being <5% (Heinemann *et al*, 2008; Sultana *et al*, 2008; Jemal *et al*, 2009). At the time of diagnosis, >85% of patients have metastatic disease, which makes surgical and medical interventions largely ineffective (Matsuno *et al*, 2004). One of the reasons for the poor outcome of PC is the lack of early detection markers and limited efficacy of existing treatment regimens. Therefore, there is an urgent need to understand the pathogenesis of PC in order to discover early detection marker(s), novel molecular targets and new therapeutic strategies.

Recent advances in molecular genetics have revealed a compendium of genetic lesions associated with the progression and metastasis of PC (Hezel *et al*, 2006). Of these mutations, *Kras* is found to be mutated in almost all cases (75–90%) of PC and represents an early event in the development and progression of this malignancy (Almoguera *et al*, 1988; Shibata *et al*, 1990; Caldas and Kern, 1995; Dergham *et al*, 1997; Moskaluk *et al*, 1997; Wang *et al*, 2002). *Kras* is a member of the highly homologous *Ras* family of proteins and has potent transforming ability (Barbacid, 1987). It is a 21 kDa size monomeric membrane-localised guanine nucleotide (GTP/GDP)-binding protein. A wide variety of extracellular stimuli can activate *Kras*, and the activated form, in turn, activates a cascade of signals that ultimately regulate cell growth, differentiation (McCormick, 1989) and apoptosis. Mutations in *Kras* occur most frequently at codon 12 (Gonzalez-Cadavid *et al*, 1989; Grunewald *et al*, 1989; Mariyama *et al*, 1989; Nagata *et al*, 1990; Shibata *et al*, 1990; Van Laethem *et al*, 1995), and less frequently at codons 13 and 61 (Motojima *et al*, 1991, 1993; Caldas and Kern, 1995). All of these mutations can abolish the intrinsic ability of the Kras protein to hydrolyse GTP, resulting in continuous stimulation of cell proliferation (Barbacid, 1987; Ellis and Clark, 2000).

Recently, several approaches, including short interfering RNAs, antibodies, mutant Kras-specific peptide inhibitors, adenoviruses expressing antisense Kras, dominant-negative Kras, antisense oligonucleotides and different drugs have been investigated for their ability to target the mutant form of Kras (Aoki *et al*, 1995, 1997; Adjei, 2001). The major drawback of dominant-negative Kras and pharmacological Kras inhibitors is the lack of specificity (Kohl *et al*, 1994; Feig, 1999; Bolick *et al*, 2003; de Bono *et al*, 2003), whereas the antisense oligonucleotides downregulate the wild-type Kras, which is essential for the normal function of all cells in the body. RNA interference (RNAi) has become a novel approach to target the mutant form of this oncogene specifically (Devi, 2006; Gaither and Iourgenko, 2007). Kras is a potent tumour initiator as evidenced by observations that activating mutations in Kras (G12D) are required for the development of pancreatic intraepithelial neoplasms (PanINs), which precede invasive adenocarcinoma (Aguirre *et al*, 2003). There is also evidence to suggest that several point mutations in codon 12 can result in constitutive activation of Kras (Karapetis *et al*, 2008). A point mutation (GGT → GAT) resulting in a single amino-acid change from glycine to aspartic acid in codon 12 (Kras^G12D^) is observed in many cases of PC as well as in many PC cell lines (Hohne *et al*, 1992). Various studies have shown the role of mutant Kras^G12D^ in enhanced cell proliferation and transformation of normal pancreatic epithelial cells (Hingorani *et al*, 2003; Tuveson *et al*, 2004); however, its function in the late stage of PC progression remains unknown.

The objective of this study was to investigate the role of mutant Kras^G12D^ allele in PC by knockdown of this allele in the highly metastatic PC cell lines CD18/HPAF and ASPC1, followed by examination of the effects on cellular functions (through *in vitro* and *in vivo* functional studies) and intracellular signalling cascades. Altogether, our data indicate that silencing of Kras^G12D^ causes a significant reduction in the motility, invasion and metastatic potential of PC cells. This is done through an upregulation of E-cadherin and downregulation of Snail, *δ*EF1 and ETV4 transcription factors, and signalling pathways such as Akt, FAK and ERK1/2.

## Materials and methods

### Cell culture, plasmid construction and transfection

CD18/HPAF, Capan-1, ASPC-1 cells were cultured in DMEM, whereas BXPC-3 cells were grown in RPMI and HPDE cells in Keratinocyte media, respectively, supplemented with 10% fetal calf serum and antibiotics (penicillin and streptomycin 100 *μ*g ml^–1^). The pSUPER.retro.puro vector was digested with *Bgl*II and *Hin*dIII restriction enzymes and dephosphorylated with calf intestinal alkaline phosphatase (CIAP). Two complementary oligonucleotides, *5′*-GATCCCCGTTGGAGCTGATGGCGTAG*TTCAAGAGA*CTACGCCATCAGCTCCAACTTTTTGGAAA-3′ and 5′-AGCTTTTCCAAAAAGTTGGAGCTGATGGCGTAG*TCTCTTGAA*CTACGCCATCAGCTCCAACGGG 3′, corresponding to the mutant *Kras*^*G12D*^ gene with *Bgl*II and *Hin*dIII sites were synthesised, annealed, phosphorylated and ligated into the digested pSUPER vector (Restriction sites at 3′ and 5′ ends are underlined while the sequence of the hairpin loop sequence is indicated by underline italics). The presence of the insert was confirmed by sequencing and digestion with *Eco*RI and *Hin*dIII restriction enzymes. The shRNA construct (pSUPER-Kras^G12D^) was transfected in the phoenix cells, a packaging cell line that produces high-viral titer in culture using Lipofectamine 2000 transfection reagent (Invitrogen, Carlsbad, CA, USA). At the same time, the phoenix cells were also transfected with pSUPER vectors bearing scramble oligonucleotide sequence. After 24hr, CD18/HPAF PC cells were seeded in 6-well plates at 5 × 10^4^ cells per well and grown to 60% confluence in DMEM without serum medium. The media supernatant was collected from phoenix cells after 48 and 72hr post-transfection and the viral supernatant was used to infect the sub-confluent cultures of CD18/HPAF PC cells after addition of 4mg mL^−1^ polybrene. Pooled populations of stable oncogenic Kras knockdown and control (ShRNA, Scramble) cells were selected by puromycin (5 *μ*g ml^–1^) containing 10% DMEM medium. The ASPC1 and BXPC3 cells were transiently knocked down for oncogenic shKras allele by using the aforementioned vector constructs (pSUPER-Kras^G12D^ and Scramble vectors). The protein was isolated from ASPC1-shKras as well as scramble controls after 48 h of transient transfection and it was analysed for downstream signalling molecules.

### Quantitative real-time PCR

Total RNA was isolated and the cDNA was synthesised by reverse transcription as described previously (Moniaux *et al*, 2008). The real-time primers for Kras^G12D^ were designed as described by Gupta *et al* (2005) by keeping the mutation at the 3′ end of the forward primer and an additional base mutation was also included before the Kras mutation in order to amplify the Kras mutant allele selectively (Gupta *et al*, 2005). For all other genes, the primers were designed using Primer 3 software (Supplementary Table 1). Real-time PCR was performed on Roche 480 Real-Time PCR System (Indianapolis, IN, USA). Real-time PCR reactions were performed in triplicate and template controls (NTCs) were run for each assay under the same conditions. PCR was then performed in 10 *μ*l reaction containing 5 *μ*l 2 × SBYR green Master Mix, 3.2 *μ*l of autoclaved nuclease free water, 1 *μ*l diluted RT product (1 : 10) and 0.4 *μ*l each of forward and reverse primers (5 pmol) for Kras mutation (F-5′-ACTTGTGGTAGTTGGAGCAGA-3′ and R-5′-TTGGATCATATTCGTCCACAA-3′). The cycling conditions comprised: 95 °C for 10 min, followed by 40 cycles of 95 °C for 15 s and by 58 °C for 1 min. Gene expression levels were normalised to the level of *β*-actin expression, which we have shown to be unresponsive to Kras mutation, and were reported relative to mutant Kras expression level in the scramble RNA-transfected cells.

### Immunoblot analysis

Immunoblot analysis was done as described previously (Moniaux *et al*, 2008). The primary antibodies for the activated form and total FAK, Kras, cyclins D1, E and A and NF-*κ*B were obtained from Santa Cruz Biotechnology (Santa Cruz, CA, USA), cMyc and p27^Kip1^ from Epitomics (Burlingame, CA, USA), activated and total Akt and ERK, Caspase-3 and Cleaved-Caspase-9 from Cell Signaling (Danvers, MA, USA), matrix metalloproteinase-9 (MMP-9) and E-cadherin were gifts from Dr Rakesh Singh and *β*-actin was from Sigma Aldrich (St Louis, MO, USA).

### Growth kinetics, clonogenicity and apoptosis assays

Cells (1.0 × 10^4^ cells per 3 ml of medium containing 1.0% FBS) were seeded in six-well plates and allowed to grow for different time intervals. The growth of the cells was monitored by counting the number of viable cells on a Vi-CELL (Boulevard, CA, USA) counter every day for 8 days. The cell population doubling time (*T*_d_) was calculated during the exponential growth phase (96–144 h) using the following formula: *T*_d_ = 0.693,t/log ,(*N*_*t*_/*N*_*0*_), where *t* is the time difference (in h), *N*_t_ is the cell number at time *t* (144 h) and *N*_0_ is the cell number at the initial time (96 h) (Zhang *et al*, 2002). To assess clonogenic potential, the cells were trypsinised and either plated in 0.3% agarose with a 0.5% agarose underlay (1 × 10^3^ cells per well in 24-well plate) for assessment of anchorage-independent growth or on plastic coated petri dishes for anchorage-dependent respectively. The number of foci >100 *μ*m was counted after 14 days. Apoptosis was measured by Annexin V FITC staining as described previously (Chaturvedi *et al*, 2007). This assay is based on the principle that during the process of apoptosis, phosphatidyl serine (normally localised on the inner leaflet of the plasma membrane) is flipped out. Annexin V has a high affinity for phosphatidyl serine and binds to it. However, at this stage (called early apoptosis), the cell membrane is still intact and hence propidium iodide (PI) is excluded from these cells. Therefore, early apoptotic cells are defined by a positive staining for Annexin V and a negative staining for PI.

As apoptosis progresses, the cell membrane permeability increases, leading to increased entry of PI that binds to the DNA. Hence, cells during the later stage of apoptosis are positive for both Annexin V and PI. A similar process also takes place during necrosis, making it impossible to distinguish late apoptosis from necrosis with this assay.

### Tumourigenicity assay

Subconfluent cultures of CD18/HPAF-derived clones were trypsinised and washed with phosphate-buffered saline. Cell viability was determined by Trypan blue staining and single-cell suspensions of >90% viability was used for the orthotopic injections. The cells were resuspended in a normal saline (NS) solution at a concentration of 5 × 10^4^ cells per 50 *μ*l. Immunodeficient mice were purchased from the Animal Production Area of the National Cancer Institute-Frederick Cancer Research and Development Center (Frederick, MD, USA). The mice were treated in accordance with the Institutional Animal Care and Use Committee (IACUC) guidelines. The orthotopic implantation was performed as previously described (Choudhury *et al*, 2004). All mice were killed after 21 days of implantation. The presence of metastatic lesions in different organs was determined thorough gross inspection and histological analysis. Pancreatic tumours were excised, weighed and measured.

### Motility and invasion assay

For motility assays, 1 × 10^6^ cells suspended in serum-free medium were plated in the top chamber of polyethylene teraphthalate membranes (six-well insert, pore size 8 *μ*m) (Becton Dickinson, Franklin Lakes, NJ, USA). Then, 2 ml of 10% serum-containing medium was added to the lower chamber of the well and the cells were allowed to migrate for 22 h under chemotactic drive. After incubation, the cells that did not migrate through the pores in the membrane were removed by scraping the membrane with a cotton swab. The migrated cells on the lower side of the membrane were stained with Diff-Quick cell stain kit (Dade-Behring Inc., Newark, DE, USA) and photographed in 10 random fields of viewed at × 100 magnification. Cell numbers were counted and expressed as the average number of cells per field of view. For invasion assay, cells (1 × 10^6^) were seeded on Matrigel-coated membrane inserts (BD Biosciences, Bedford, MA, USA). The bottom chamber contained 2.0 ml of serum-supplemented medium as a chemoattractant. After incubation for 22 h at 37 °C, the cells that had invaded through the Matrigel-coated membrane were fixed and stained using a Diff-Quick reagent kit. After air drying the membrane, the cells were counted at a magnification of × 10 in 10 random fields of view under a microscope. Three independent experiments were done in each case. The data were represented as the average of the three independent experiments with the standard error of mean (s.e.m.).

### Oligonucleotide array gene expression analysis

Human oligonucleotide array containing probes for 39 200 genes was constructed at the Microarray Core Facility of University of Nebraska Medical Center. Total RNA was isolated from shK-ras and K-ras scramble transfected CD18/HPAF cells by Qiagen RNEasy kit (Qiagen Sciences, Valencia, CA, USA) according to the manufacturer's directions. The procedure for the microarray hybridisation and the subsequent analysis has been previously described by us (Chaturvedi *et al*, 2007).

### Statistical analysis

For analysis of microarray data, a gene chip containing 39 200 genes was used. The data were normalised using BRB Array Tools. Random-variance paired *t*-tests were used to determine which genes are differentially expressed between tumour samples and the normal samples. The random-variance paired *t*-test allows for sharing information among genes about variation without assuming that all genes have the same variance, which gives a more accurate estimate of the variability when sample sizes are small (2). A significance level of 0.001 was selected to help limit the false discovery rate (FDR) due to multiple comparisons. The FDR was limited to <10%. Parametric data were compared using the two-tailed Student's *t*-test, whereas nonparametric data were analysed using a two-way ANOVA or *χ*^2^ test. Data were analysed using the Medcalc for Windows version 9·6·4·0 software (MedCalc Software, Broekstraat, Mariakerke, Belgium). A *P*-value of <0.05 was considered significant.

## Results

### Targeting of mutant Kras^G12D^ allele by stable expression of Kras^G12D^ shRNA leads to decreased oncogenic Kras expression

**Figure 1:**
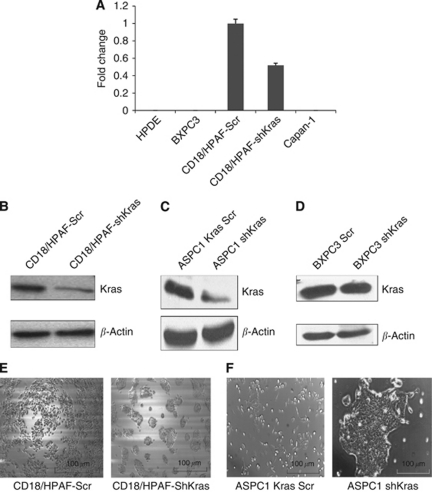
Strategy for shRNA-mediated sequence-specific silencing of oncogenic Kras in CD18/HPAF cells. (**A**) Real-time PCR analysis using primers that specifically amplify mutated codon 12 but not wild-type allele. CD18/HPAF-shKras^G12D^ pooled population cells show a 60–70% decrease in the expression of mutant Kras (G → D) allele at the mRNA level compared with the control population. No amplification was observed in BXPC3, HPDE and Capan-1 cells, which are negative for the K-ras^G12D^ mutant allele. (**B**, **C**) Western blot analysis of the CD18/HPAF (or ASPC-1) shKras^G12D^, CD18/HPAF (or ASPC-1) Scr pooled population shows significant inhibition in total Kras protein expression, respectively. (**D**) Specificity of Kras shRNAs for Kras^G12D^ allele: BXPC3 cells transiently transfected with pSUPER retro shKras or Kras-Scr revealed no significant difference in total levels of Kras protein by immunoblotting. *β*-Actin was served as a loading control. (**E**, **F**) A morphological comparison between the CD18/HPAF (or shASPC-1) shKras^G12D^ and CD18/HPAF (or ASPC-1) Kras-Scr cells. The Kras knockdown CD18/HPAF (or shASPC-1) cells grow as aggregates compared with the scramble cells.

To target the *Kras*^*G12D*^ mutant allele, siRNA oligos were designed that were 64 nucleotides long and covered with a point mutation in codon 12 (G → D) of the *Kras* gene. They were cloned into the pSUPER RETRO mammalian expression vector. Similarly, a scramble expression vector construct (pSUPER Kras-Scr) was made using scramble shRNA oligonucleotides. The resultant constructs (pSUPER-shKras^G12D^ and pSUPER Kras-Scr) were transfected into CD18/HPAF pancreatic adenocarcinoma cells. Pooled populations of CD18/HPAF-shKras^G12D^ and CD18/HPAF-Kras-Scr were selected for puromycin resistance. The effective inhibition of the mutant Kras^G12D^ allele was determined by real-time PCR using a primer set that selectively amplifies the mutated Kras allele but not the wild-type (WT) allele. RNAs isolated from BXPC3, HPDE and Capan-1 cells were used as a control as it is known that BXPC3 and HPDE cells express only the WT alleles and Capan-1 has a G12V mutation instead of the G12D mutation. The CD18/HPAF-shKras cells had a significantly decreased expression of Kras^G12D^ mRNA compared with the CD18/HPAF-Kras-Scr cells (Figure 1A). Furthermore, the expression of total Kras protein was reduced in the CD18/HPAF-shKras^G12D^ cells compared with Kras-Scr-transfected cells (Figure 1B). Similar results were observed with transient knockdown of the oncogenic Kras allele in ASPC1 cells (Figure 1C).

In order to confirm the specificity of the oligos, we transiently transfected BXPC3 cells (Kras^G12D^ negative) with pSUPER-shKras^G12D^. The pSUPER-Kras-Scr vectors revealed no significant decrease in Kras total protein between the BXPC3 Scr and BXPC3 shKras (Figure 1D).

### Silencing of mutant Kras^G12D^ allele leads to altered morphology, decreased growth rate and reduced clonogenicity of pancreatic cancer cells

**Figure 2:**
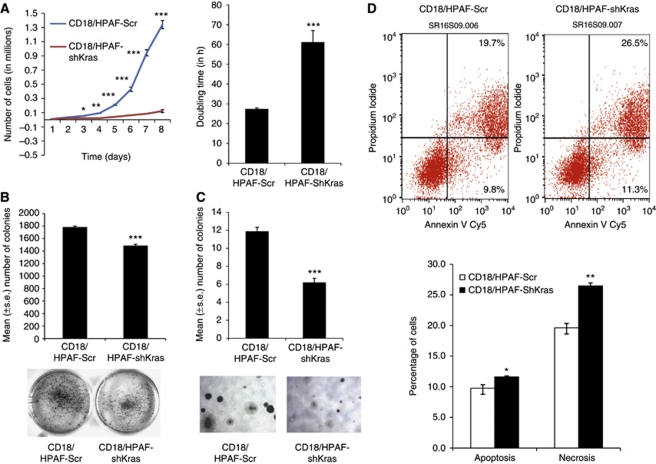
(**A**) Growth kinetics of shKras pooled population and vector control (scramble) cells. A total of 10 000 cells were plated in six-well plates in DMEM media containing 1.0% FBS. Cells were counted every 24 h for 8 days and a growth curve was plotted. The shKras clones showed reduced cell growth compared with the vector control cells. Doubling time of these cells was calculated at 96–144 h; the shKras clone showed a higher doubling time compared with the vector control cells (^*^P<0.05, ^**^P<0.001, ^***^P<0.0001). (**B**, **C**) Anchorage-dependent (**B**) and -independent (**C**) analysis for short-term colony formation in CD18/HPAF-shKras^G12D^ and scramble cells. Statistical analysis revealed a significant variation (anchorage-independent (^***^P<5.002E–08) and anchorage-dependent conditions (^***^P<0.0001)) between the two populations. (**D**) Annexin V and propidium iodide staining analysis by flow cytometry to identify apoptosis in CD18/HPAF-shKras^G12D^ and scramble cells. The statistical analysis showed significant variation in necrotic cells (^**^P=0.002) and apoptotic cells (^*^P=0.05).

The morphology and growth rates of the pooled populations of CD18/HPAF-shKras and CD18/HPAF-Kras-Scr cells were monitored after inhibiting oncogenic Kras (mutant allele) in tumour cells. The CD18/HPAF-shKras cells showed a tendency to grow as clumps when compared with the CD18/HPAF-Kras-Scr cells (Figure 1E). Similar growth pattern was observed with silencing of mutant Kras^G12D^ allele in ASPC1 cells (Figure 1F). A growth curve was plotted to determine the effect of *Kras* knockdown on the cell doubling time. Calculation of population doubling time during the exponential phase (96–144 h) demonstrated a significant (*P*<0.0045) increase in cell doubling time in the CD18/HPAF-shKras cells (61.0 h) compared with scramble siRNA-transfected cells (27.0 h; Figure 2A). Furthermore, on the last day (day 8), there was nearly a 90% reduction in the number of cells in the CD18/HPAF-shKras group when compared with the CD18/HPAF-Kras-Scr group (Figure 2A).

The effect of silencing oncogenic Kras^G12D^ expression on the clonogenic properties of CD18/HPAF cells was studied in anchorage-independent and anchorage-dependent conditions. We observed that the CD18/HPAF-shKras^G12D^ cells had a significantly reduced ability to divide as evidenced by the reduction in the number of colonies formed in both anchorage-independent (*P*<0.0001) and anchorage-dependent conditions (*P*<0.0001; Figures 2B and C). Analysis of PI- and Annexin V-positive cells by flow cytometry indicated a significant increase in the number of apoptotic cells (*P*<0.05; with Annexin V-positive but PI-negative staining) and late apoptotic/necrotic cells (*P*<0.002; with Annexin V-positive and PI-positive staining) in CD18/HPAF-shKras cells when compared with CD18/HPAF-Kras-Scr cells (Figure 2D).

### Oncogenic Kras knockdown results in an inhibition of cell motility and invasion

**Figure 3:**
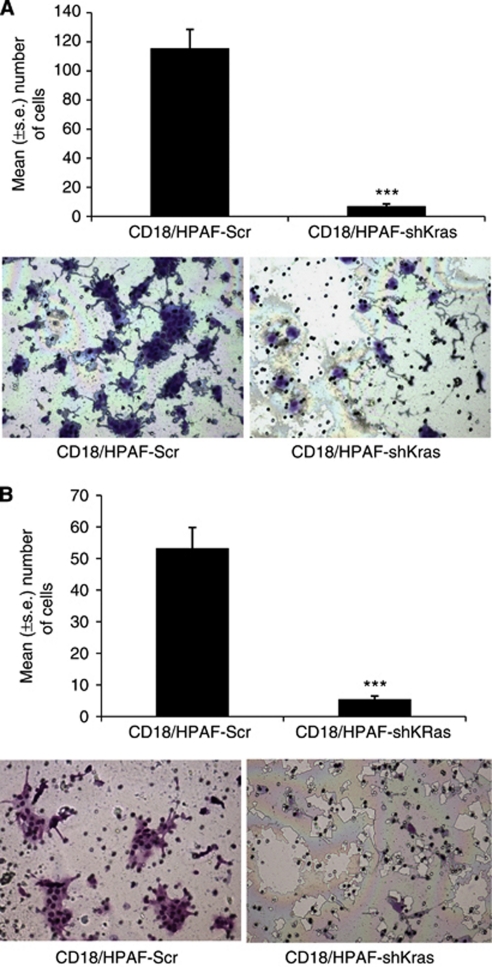
Effect of oncogenic K-ras on cell motility and invasive behaviour of CD18/HPAF cells. Cells (1 × 10^6^) were seeded on non-coated or Matrigel-coated membranes for motility (**A**) and invasion (**B**) assays, respectively, and incubated for 24 h. Medium containing 10% fetal bovine serum in the lower chamber was used as a chemoattractant. The cells that migrated through the membrane were stained and photographed under bright-field microscopy (magnification × 10). The number of cells that migrated through the membrane was determined by averaging 10 random fields of view. The data are expressed as the number of cells per field of view and is the average of three independent experiments. Error bars indicate s.e. of the average (^***^P<0.0001). The total numbers of CD18/HPAF-shKras^G12D^ cells that migrated and invaded were reduced by six- and ten-fold respectively as compared with the vector control cells.

Several studies have reported that invasive and metastatic properties of tumour cells are partly influenced by their phenotypic characteristics such as motility and invasion. Silencing of oncogenic *Kras*^*G12D*^ leads to a significant (*P*<0.0001) reduction in cellular motility and invasive ability (∼6- and 10-fold, respectively) in CD18/HPAF cells (Figures 3A and B).

### Selective inhibition of oncogenic Kras^G12D^ in pancreatic cancer cells results in the suppression of tumourigenicity and metastasis

**Figure 4:**
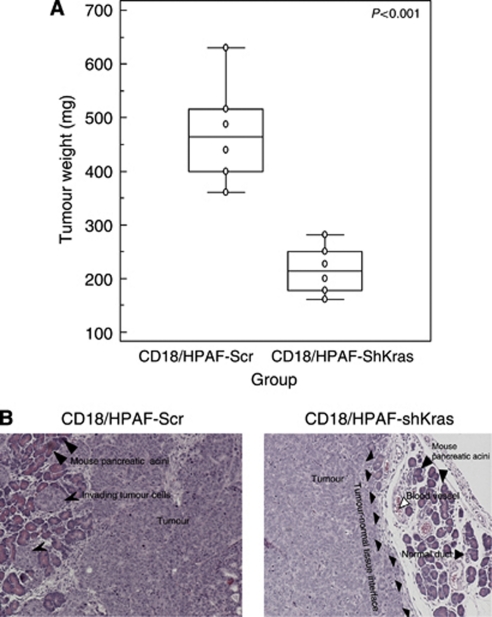
Effect of oncogenic Kras knockdown on size of orthotopically grown primary tumours. (**A**) The mean weight of tumours formed by CD18/HPAF-shKras cells was significantly less than that formed by the scramble cells (*P*<0.001). (**B**) Haematoxylin and eosin-stained sections of orthotopic tumours demonstrating the aggressive invasion of the normal pancreatic tissue by the CD18/HPAF-Kras-Scr cells (left). In comparison, CD18/HPAF-shKras^G12D^ cells showed significantly decreased invasiveness (as evidenced from a clear demarcation between the edge of the tumour and the healthy tissue) (right).

To examine the effect of oncogenic Kras knockdown *in vivo,* a pooled population of CD18/HPAF-shKras^G12D^ and CD18/HPAF-Kras-Scr cells was orthotopically implanted into the pancreas of nude mice. The animals were killed at 21 days post-implantation and the pancreatic tumours were removed and weighed. We carried out haematoxylin and eosin staining (Figures 4A and B). Liver, lung, diaphragm, intestine, kidney and mesenteric lymph nodes were examined for the presence of metastatic lesions. A primary pancreatic tumour and metastatic lesions in the spleen and on the intestinal wall were found in all the mice implanted with CD18/HPAF-Kras-Scr cells. In this group, some animals also had metastasis in the liver (*n*=2) and/or kidney (*n*=1). In contrast, animals injected with CD18/HPAF-shKras cells had significantly smaller tumours (*P*<0.001), and had fewer or no metastatic lesions (Table 1).

### Effect of Kras^G12D^ silencing on downstream signalling

**Figure 5:**
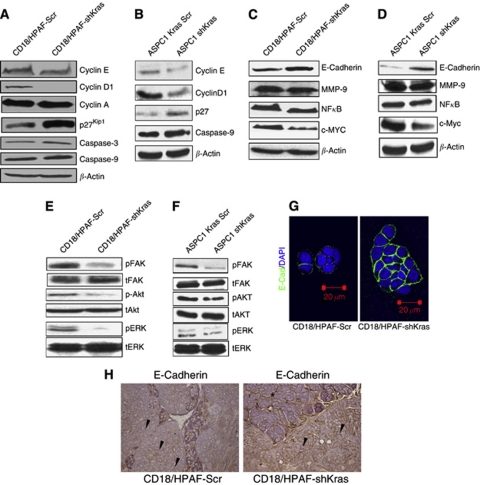
Western blot analysis comparing the expression of key molecules involved in cell proliferation (c-myc, cyclins A, D1, E and p27), apoptosis (caspase-3 and 9), metastasis and invasion (E-cadherin, MMP-9, NF-*κ*B, phospho and total FAK, phosphor and total Akt and phosphor and total ERK-1/2) in CD18/HPAF (**A**, **C**, **E**) and ASPC-1 (**B**, **D**, **F**) Scr *vs* shKras^G12D^ cells. (**H**) Immunohistochemical analysis of primary tumours developed following orthotopic implantation of CD18/HPAF-shKras^G12D^ and scramble cells in immunodeficient mice. Tumour sections were stained for E-cadherin using specific anti-mouse monoclonal antibody (original magnification × 100). Tissue derived from CD18/HPAF-shKras^G12D^ tumours showed a stronger membrane staining for E-cadherin (arrowheads). (**G**) Confocal analysis showed increased E-cadherin expression on the cell membrane in CD18/HPAF-shKras^G12D^ cells compared with scramble cells (^*^Normal mouse pancreatic tissue, primary tumour with E-cadherin expression).

*Kras* mutation has previously been reported to be associated with the upregulation of cyclins D and E and downregulation of p27^kip1^ (Fan and Bertino, 1997). As shown in Figures 5A and B, the sequence-specific knockdown of the activated Kras allele led to a decreased expression of cyclins D1 and E in the CD18/HPAF-shKras and ASPC1-shKras cells in comparison with the Kras-Scr-transfected cells, whereas no change was observed in cyclin A levels. Similarly, the expression of p27^kip1^, caspase-3 and cleaved caspase-9 was also increased in shKras^G12D^ transfected cells compared with the Kras-Scr-transfected CD18/HPAF and ASPC1 cells. Inhibition of the *Kras*^*G12D*^ allele expression resulted in a significant decrease in the activation of downstream signalling molecules, including phospho-ERK1/2, phospho-Akt and phospho-FAK, MMP-9, c-Myc, NF-*κ*B in the shKras-transfected cells compared with Kras-Scr-transfected cells (Figures 5C–F). The level of total ERK-1/2, Akt and total FAK, however, remained unchanged. Furthermore, immunoblot analysis also revealed an increased expression of E-cadherin in the CD18/HPAF-shKras and ASPC1-shKras cells compared with the scrambled population (Figures 5C and D). In agreement with these results, immunofluorescence analysis also revealed an increase in E-cadherin expression and membrane localisation in CD18/HPAF-shKras-transfected cells when compared with CD18/HPAF-Kras-Scr knockdown cells (Figure 5G). Furthermore, immunohistochemical analysis of the primary orthotopic tumour sections (from the orthotopically implanted mice) revealed an increased E-cadherin expression in the CD18/HPAF-shKras tumours compared with the scramble vector-transfected cells (Figure 5H).

### Alteration in signalling pathways because of knockdown of mutant Kras allele in CD18/HPAF cells

**Figure 6:**
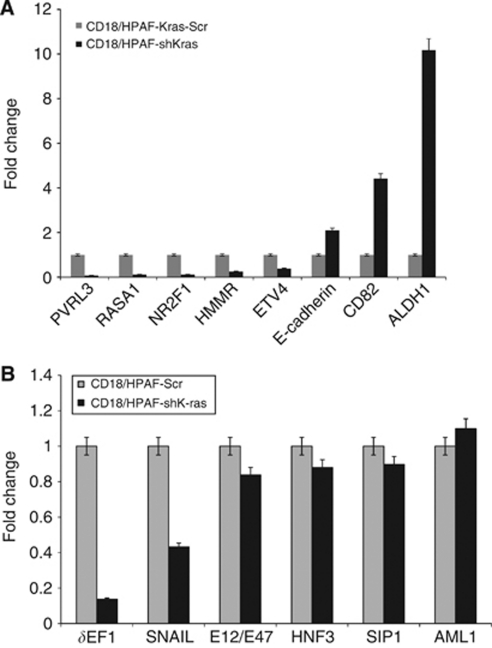
Quantitative real-time PCR analysis. (**A**) The expression of selected upregulated and downregulated genes (identified by global microarray analysis) was validated by Q-RT–PCR using specific primers. (**B**) Real-time PCR analysis of the expression of transcription factors known to downregulate E-cadherin expression.

In order to identify the pathways dysregulated in PC cells because of the knockdown of the mutant Kras allele, we compared the gene expression profiles of CD18/HPAF-shKras and scramble cells by global microarray analysis. The microarray analysis revealed that many genes were significantly up- or down-regulated more than two-fold in CD18/HPAF-shKras cells compared with the scrambled cells (Supplementary Tables 2 and 3). Notably, the functional classes of genes affected by Kras silencing included tumour suppressors (*HMMR*, *CAV1* and *BHLHE41*), cell adhesion molecules (*CDH1*, *LGALS4* and *PVRL3*), genes regulating cellular motility and invasion (*ETV4*, *NT5E* and *ALDH1A1*), cell growth (*GCNT3*), cell cycle (*HPGD*, *CDKN1A* and *CAV1*), metastasis (*CD82*) and signal transduction (*TM4SF4* and *NR2F1*). Out of these pathways, the pathway modulated by the transcription factor ETV4, SNAIL and *δ*EF1 appeared to be highly perturbed. Using the Ingenuity Pathway Analysis (Ingenuity Systems, Mountain View, CA, USA) software, the differentially expressed genes were grouped into several gene networks (Supplementary Figure 1). Some of the differentially expressed genes were validated by real-time PCR (Figure 6A). The real-time PCR also showed a reduced expression of SNAIL and *δ*EF1 transcription factors in Kras knockdown cells compared with control cells (Figure 6B). The gene ontology-based clustering analysis revealed that many of the genes differentially regulated upon silencing of Kras were involved in cell adhesion and metastasis. In summary, the microarray analysis suggests that Kras signalling is important in the process of PC metastasis and may crosstalk with other signalling pathways.

## Discussion

Cancer development involves a multistep process in which tumour cells acquire various genetic and epigenetic changes to grow and metastasise to distant organs. Investigation of the molecular genetics of pancreatic adenocarcinoma has revealed a specific pattern of genetic lesions that occur during the initiation and progression of PC (Aguirre *et al*, 2003; Hezel *et al*, 2006). Out of these, mutations in the *Kras* gene are reported to be an early event, being observed in virtually all cases of PC (75–95% Almoguera *et al*, 1988; Wang *et al*, 2002). These mutations in Kras have an important role in the initiation and progression of PC. During the later part of the disease, other genetic and epigenetic alterations occur in *EGFR*, *HER2*, *p16*^*Ink4a*^, *p53*, *Smad/DPC4*, and other genes (Aguirre *et al*, 2003; Hezel *et al*, 2006) that facilitate the development of pancreatic adenocarcinoma and, subsequently, its metastasis (Aguirre *et al*, 2003; Hingorani *et al*, 2005; Bardeesy *et al*, 2006; Hezel *et al*, 2006). The dominant nature of the mutant Kras allele results in the cells exhibiting a transformed ability even when a single allele of mutant Kras is expressed. Consequently, inhibition of the oncogenic Kras allele expression in human cancers is a promising approach for tumour-specific gene therapy (Friday and Adjei, 2005). Previous studies have shown that Kras^G-12D^ has lower intrinsic GTPase activity than WT Kras. Furthermore, this mutant is also insensitive to p-120-GAP (Bollag *et al*, 1996), leading to constitutive activation of Ras-mediated downstream signalling pathways in cells expressing the mutant Kras allele. Therefore, studies on the molecular and cellular functions associated with this mutant hold paramount importance for therapeutic purposes.

In this study, the expression of the mutated Kras^G12D^ allele was selectively inhibited by shRNA, specifically targeting the mutant allele in CD18/HPAF and ASPC-1 PC cells. Subsequently, we studied the effect of Kras^G12D^ silencing on the function of PC cells *in vitro* (motility, invasion and clonogenicity) and *in vivo* (tumourigenesis and metastasis) and its impact on downstream signalling pathways by using pooled populations. In the case of CD18/HPAF cells, suppression of the oncogenic Kras^G12D^ allele led to a significant reduction in their tumourigenic and metastatic potential *in vivo*. Microarray analysis identified several genes associated with cell growth, proliferation and metastasis that were significantly altered in the CD18/HPAF-Kras^G12D^ knockdown cells.

The stable expression of a shRNA targeting the mutant Kras^G12D^ allele led to a decreased expression of the mutant allele at the mRNA level in CD18/HPAF-shKras cells (Figure 1A). The total Kras protein expression was also significantly decreased in the CD18/HPAF-shKras ASPC1-shKras cells (Figures 1B and C). These results suggest that siRNAs can serve as powerful tools for sequence-specific inhibition of an oncogenic mutant allele. Our study also corroborates previous studies in human (Capan-1 Kras^G12V^) and murine (C26 colorectal cells, Kras^G12D^) cells, wherein the stable knockdown of mutant Kras allele resulted in a reduced expression of both the oncogenic and total Kras protein and mRNA levels (Brummelkamp *et al*, 2002; Smakman *et al*, 2005).

It is now clearly established that activating mutations in Kras are found in the majority of PCs and that these mutations, along with other genetic lesions, contribute to the increased aggressiveness of the tumour (Almoguera *et al*, 1988; Dergham *et al*, 1997; Wang *et al*, 2002; Aguirre *et al*, 2003; Hezel *et al*, 2006). Therefore, the Kras has emerged as an attractive target for the therapy of PC (Friday and Adjei, 2005). In this study, the sequence-specific inhibition of mutant Kras^G12D^ allele resulted in a significant decrease in tumour cell growth and an altered morphology and clonogenic ability (Figures 1E and 1F and 2A–C). This was expected because of the established role of Kras in cell proliferation and cell survival (Fan and Bertino, 1997; Hingorani *et al*, 2003; Tuveson *et al*, 2004). Activated Kras regulates the cell cycle through activation of ERK and Akt signalling pathways (Wang *et al*, 2009) and induction of cyclin D1, a protein important for progression from the G1 to the S phase (Stacey, 2003). Furthermore, it also has an antiapoptotic effect mediated by activation of the PI3-K/Akt pathway (Downward, 2004). In our study, inhibition of mutant Kras resulted in suppression of the ERK and Akt pathways (Figures 5E and F). Similarly, there was a significant decrease in cellular levels of c-Myc, NF-*κ*B, cyclins D1 and E and an increase in caspase-3, 9 and p27^kip1^ levels in the CD18/HPAF-shKras and ASPC1-shKras cells (Figures 5A–D). This result suggests that the constitutively active Kras allele is important for survival, proliferative ability, motility and invasiveness of PC cells. Thus, the Kras^G12D^ allele appears to have an important role in regulating key cellular processes in PC cells, reinforcing its importance as a target for anticancer therapy.

Tumour metastasis comprises a series of distinct and sequential steps, involving the growth of the tumour locally, invasion by transmigration through basement membrane and nontumor host tissue, intravasation into blood vessels, dissemination and survival in the bloodstream and finally extravasation and re-establishment at distant sites (Chambers *et al*, 2002). It requires a series of cellular processes to occur, including phenotypic changes, loss of the cell–cell and cell–extracellular matrix (ECM) interactions and increased cellular motility. In this study, we observed a decrease in cell motility and invasion in the Kras knockdown cells (pooled population) associated with decreased activation of the Akt pathway (Figures 3A, B, 5E, F). Cell motility has a key role in tumour cell invasion into the surrounding non-tumour tissues and is a major determinant of the aggressive nature of a tumour cell. The Ras pathway has also been implicated in cytoskeletal rearrangements, altering the expression of integrins and cell migration through activation of the PI3-K/Akt pathway (Potempa and Ridley, 1998; Okudela *et al*, 2004; Fleming *et al*, 2005).

Inhibition of Kras^val12^ by shRNA has been previously shown to decrease tumourigenicity in Capan-1 cells upon subcutaneous implantation (Brummelkamp *et al*, 2002) and in murine C26 colorectal cancer cells *in vitro.* The Kras knockdown cells formed fewer tumours and did not cause morbidity (Smakman *et al*, 2005). We also noted that inhibition of Kras^G12D^ resulted in a significant decrease in the tumourigenic and metastatic potential of CD18/HPAF (Figure 4A and Table 1). However, there was no decrease in the incidence of tumours with 100% of the animals injected with the CD18/HPAF-shKras^G12D^ cells forming tumours. The possible explanations for this observation include the presence of heterogeneous cells in the pooled clonal population and the partial compensation of the anti-tumourigenic effect of Kras^G12D^ knockdown by other unknown signalling pathways. Although these earlier studies indicated that oncogenic Kras has a key role in the proliferation of PC cells, its role in regulating metastasis of PC has remained largely unexplored. In order to determine the role of activated Kras in metastasis, we utilised an orthotopic model that revealed a significant inhibition of metastasis upon selective silencing of Kras^G12D^ (Table 1). Our results suggest that the constitutively active form of Kras not only regulates tumour cell growth, but it also has an important role in modulating the invasive nature of the malignant cells.

Tumour cell invasion through the ECM and tissue barriers requires the combined effects of increased cell motility and proteolytic degradation. We observed decreased levels of activated FAK (pY925) in the CD18/HPAF-shKras and ASPC1-shKras cells when compared with that in the scramble cells (Figures 5E and F). The decreased activation of FAK may be responsible for the reduced motility observed in the CD18/HPAF-shKras cells compared with scramble cells similar to that reported in an earlier study (Sieg *et al*, 2000).

Additionally, the phenotypic changes associated with epithelial mesenchymal transition (EMT) include both an increased cellular motility (Thiery, 2002) and an increased production of ECM-degrading enzymes, accompanied by disruption of E-cadherin-mediated cell–cell adhesion (Takeichi, 1995; Christofori and Semb, 1999). The function of epithelial E-cadherin is altered in most epithelial tumours and can be disrupted by various genetic and epigenetic mechanisms, including modulation by signalling molecules. Loss of E-cadherin activates signals that promote tumour cell migration, invasion and dissemination (Thiery, 2002). The increased expression of E-cadherin in the Kras knockdown cells (Figures 5C, D, G, H and 6A, and Supplementary Figure 1) seems to suggest that oncogenic Kras^G12D^ can inhibit E-cadherin function partly by suppressing its expression. Furthermore, the expression of MMP-9, a key mediator of the invasive property of malignant cells (Fridman *et al*, 2003), was also decreased upon silencing of the oncogenic Kras allele. Kras-mediated ERK activation is known to induce MMP-9 that, in turn, causes cleavage of E-cadherin leading to the disruption of cell–cell contacts (Wang *et al*, 2009). Our results suggest that a MMP-9-mediated decrease in E-cadherin expression may contribute to the highly metastatic property of CD18/HPAF cells. This suggests that activated Kras contributes to the metastatic nature of CD18/HPAF cells.

To elucidate the global cellular pathways that are altered in PC cells upon downregulation of the mutant Kras allele, we conducted microarray analysis and observed that most of the genes differentially expressed in the Kras-Scr compared with the shKras cells were associated with cell proliferation, motility and metastatic behaviour of tumour cells (Figure 6A, Supplementary Tables 2 and 3). Of those genes that were altered because of silencing of oncogenic Kras, alteration in the expression of the transcription factor Snail supports Kras-mediated E-cadherin regulation. Snail has been shown to promote tumour cell invasion by either inducing the transcription of MMP-9 (Jorda *et al*, 2005) or suppression of transcription of E-cadherin (Huber *et al*, 2005). Recently, a report showed that *δ*EF-1/ZEB1 binds to the promoter of the E-cadherin and represses its expression (Eger *et al*, 2005). In the present study, real-time PCR analysis showed that the expression of both *SNAIL* and *δ*EF1/ZEB1 was decreased in the CD18/HPAF-shKras cells (Figure 6B). Altogether, these results, including the increase in E-cadherin, point to *SNAIL* and *δEF1/ZEB1*-mediated regulation of E-cadherin as a target of *Kras* in PC cells. In addition, the microarray analysis identified several differential genes associated with cell proliferation, motility and metastatic behaviour of tumour cells (Supplementary Tables 2 and 3 and Supplementary Figure 1). Taken together, the results of our study provide an insight into the diverse pathways altered in PC cells upon the sequence specific inhibition of the mutant Kras allele.

**Figure 7:**
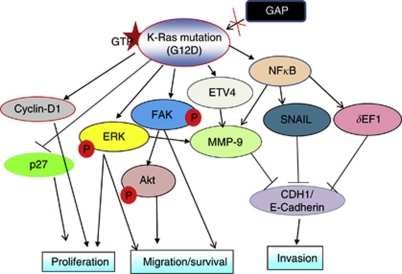
Proposed model for Kras-mediated signalling events that promote progression and metastasis of pancreatic cancer. A glycine (G) to aspartate (D) mutation in the Kras protein makes it constitutively active by making it insensitive to inactivation by GTPase-activating proteins (GAPs). The constitutively active Kras (Kras^G12D^) can then activate several pathways involved in cell proliferation, migration, invasion and metastasis of PC cells. The activated Kras upregulates cyclin D1 and downregulates the expression of p27, leading to cell proliferation. Mutant Kras-mediated activation of extracellular signal-regulated kinase (ERK) can activate downstream signalling pathways that promote cell proliferation, survival, migration and metastasis. Likewise, Kras-mediated activation of focal adhesion kinase (FAK) leads to the activation of Akt, and thus promotes cell migration and survival. The activated FAK can also directly regulate cell motility. Kras-mediated upregulation of NF-*κ*B leads to an upregulation of the transcription factors SNAIL and *δ*EF1 that, in turn, repress the expression of E-cadherin, a key molecule regulating cell migration/invasiveness. At the same time, it also upregulates ETV4 (a transcription factor) that, in turn, promotes the expression of matrix metalloproteinase-9 (MMP-9), a gelatinase that promotes cancer cell invasion.

This study was aimed at understanding the pathological role of the highly oncogenic Kras^G12D^ allele in PC. The specific silencing of the oncogenic Ras allele downregulated multiple signalling pathways that are involved in promoting cell proliferation, inhibiting apoptosis, breaking cell–cell contacts and regulating expression of protease like MMP-9. The observed inhibition of cell proliferation in the Kras knockdown cells may be mediated through the inhibition of the MAPK pathway, whereas the increase in the metastatic property of Kras^G12D^ expressing cells might be at least partly because of the activation of FAK and a reduction in the expression of E-cadherin (Figure 7). Finally, our studies demonstrated that PC cells harbouring the Kras^G12D^ mutation are dependent on Ras signalling and suggest that shRNA-mediated gene silencing could be an effective approach for selective inhibition of activated Kras. The results of our study could be useful to target novel proteins downstream of activated Kras in order to disrupt Ras-mediated oncogenic signalling pathways.

## Figures and Tables

**Table 1:** Incidence of metastases developed by orthotopic implantation of pooled populations of CD18/HPAF Scr cells and shKras clones in immunodeficient mice

**Cell type**	**Spleen**	**Liver**	**Peritoneum**	**Mesenteric lymph nodes**	**Kidney**	**Intestinal wall**
CD18/HPAF scramble	4/6 (67%)	2/6 (33%)	4/6 (67%)	4/6 (67%)	1/6 (17%)	5/6 (83%)
CD18/HPAF shK-ras	0/6 (0%)	0/6 (0%)	0/6 (0%)	1/6 (17%)	0/6 (0%)	0/6 (0%)
*P*-value (*χ*^2^ test)	0.06	0.45	0.06	0.24	1.0	0.01
