# Supplementary material for: Activated KrasG12D is associated with invasion and metastasis of pancreatic cancer cells through inhibition of E-cadherin
Source: Br J Cancer. 2011 Mar 1;104(6):1038–48. doi: 10.1038/bjc.2011.31 (PMC3065271; doi:10.1038/bjc.2011.31)
Supplement: Supplementary Table 1 [file bjc201131x1.doc]

**Supplementary Table 1: Real time PCR primers for genes used in the study**

|  | **Gene name** | **Primer sequence** | **Product size (bp)** | **Annealing temperature (°C)** |
| --- | --- | --- | --- | --- |
| 1 | CD82 | F:5'-GAGAACCTGGGCATCATCCT-3’ | 120 | 60 |
|  |  | R:5'-TTGGGGACCTTGCTGTAGTC-3’ |  |  |
| 2 | ALDH1A1 | F:5'-TTGCTATGGCGTGGTAAGTG-3’ | 118 | 60 |
|  |  | R:5'-CACTGTGACTGTTTTGACCTCTG-3’ |  |  |
| 3 | RASA1 | F:5'-GTAATGAGCGTGGTGCACAG-3’ | 116 | 60 |
|  |  | R:5'-CGAAGGCTGCTACCTGACAT-3’ |  |  |
| 4 | ETV4 | F: 5'-AAAGGCATCATGCAGAAGGT-3’ | 117 | 60 |
|  |  | R: 5'-CTCAGCCTTGAGAGCTGGAC-3’ |  |  |
| 5 | NR2F1 | F: 5'-ATCGTGCTGTTCACGTCAGA-3’ | 110 | 60 |
|  |  | R: 5'-GGGTACTGGCTCCTCACGTA-3’ |  |  |
| 6 | HMMR | F:5'-TCCTTCAAAGGCTTTTCATCA-3’ | 113 | 60 |
|  |  | R:5'-TCCATGATTCTTGACACTCCA-3’ |  |  |
| 7 | E-Cadherin | F:5’-ATGAGTGTCCCCCGGTATCT-3’ | 117 | 60 |
|  |  | R:5’-TCAGGGAGCTCAGACTAGCAG-3’ |  |  |
| 8 | SIP1 | F: 5'-TTTGACCAACGTGATTTAGCTG-3’ | 111 | 60 |
|  |  | R: 5'-TGAATTGGCATTGTTTTCCTC-3’ |  |  |
| 9 | E12/E47 | F: 5'-GAATCCCAAAGCAGCCTGT-3’ | 115 | 60 |
|  |  | E12/E47R: 5'-GTTGTGGGCTTCGCTCAG-3’ |  |  |
| 10 | δEF1 | F: 5'-GACCTCATGAGTGTGGAATCTG-3’ | 118 | 60 |
|  |  | R: 5'-AAGCGCTTTCCACATTTGTC-3’ |  |  |
| 11 | SNAIL | F:5'-TTCGGACCCACACATTACCT-3’ | 114 | 60 |
|  |  | R:5'-GCAGTGAGGGCAAGAAAAAG-3’ |  |  |
| 12 | HNF3 | F: 5'-AGTTCCCGGTGAACCAGTC-3’ | 112 | 60 |
|  |  | R: 5'-ATGCGGACTCGCTTGCTAT-3’ |  |  |
| 13 | AML1 | F: 5'-TTTCCAGTCGACTCTCAACG-3’ | 124 | 60 |
|  |  | R: 5'-GTCGGGGAGTAGGTGAAGG-3’ |  |  |
| 14 | PVRL3 | R: 5'-AAAGTGACCAATTCCCTTGG-3’ | 120 | 60 |
|  |  | R:5'-CTCGATGTCAGCAGTTGAGG-3’ |  |  |
