# Supplementary material for: Activated KrasG12D is associated with invasion and metastasis of pancreatic cancer cells through inhibition of E-cadherin
Source: Br J Cancer. 2011 Mar 1;104(6):1038–48. doi: 10.1038/bjc.2011.31 (PMC3065271; doi:10.1038/bjc.2011.31)
Supplement: Supplementary Table 2 [file bjc201131x2.doc]

**Supplementary table 2: Common up regulated genes in CD18/HPAF shKras pooled population**

| **Gene name** | **Official Symbol** | **Fold change CD18/HPAF**  **shK-ras/Scramble** | **Function** |
| --- | --- | --- | --- |
| Hydroxyprostaglandin dehydrogenase 15-(NAD) | HPGD | 9.7 | negative regulation of cell cycle |
| Aldehyde dehydrogenase 1 family, member A1 | ALDH1A1 | 7.6 | Inhibiting invasion in these pancreatic cancer cell lines |
| Deleted in malignant brain tumors 1 | DMBT1 | 6.1 | Interaction of tumor cells and the immune system |
| Transmembrane 4 L six family member 4 | TM4SF4 | 3.5 | Signal transduction |
| Glucosaminyl (N-acetyl) transferase 3, mucin type | GCNT3 | 2.8 | Cell growth and adhesion |
| Basic helix-loop-helix family, member e41 | BHLHE41 | 2.5 | Tumor suppressor and |
| Cyclin-dependent kinase inhibitor 1A (p21, Cip1) | CDKN1A | 2.3 | Regulator of cell cycle progression at G1 |
| CD82 molecule | CD82 | 2.3 | Metastasis suppressor gene |
| Lectin, galactoside-binding, soluble, 4 [ | LGALS4 | 2.1 | Cell adhesion |
| cadherin 1, type 1, E-cadherin (epithelial) | CDH1 | 1.9 | Cell adhesion |
