# Supplementary material for: Activated KrasG12D is associated with invasion and metastasis of pancreatic cancer cells through inhibition of E-cadherin
Source: Br J Cancer. 2011 Mar 1;104(6):1038–48. doi: 10.1038/bjc.2011.31 (PMC3065271; doi:10.1038/bjc.2011.31)
Supplement: Supplementary Table 3 [file bjc201131x3.doc]

**Supplementary Table 3: Common down regulated genes in CD18/HPAF shKras pooled population**

| **Gene name** | **Official Symbol** | **Fold change CD18/HPAF**  **shK-ras/Scr** | **Function** |
| --- | --- | --- | --- |
| Protease, serine, 7 (enterokinase) | PRSS7 | 0.3 | Catalytical competence via proteolytic cascades |
| RAS p21 protein activator (GTPase activating protein) 1 | RASA1 | 0.3 | Suppressor of RAS function |
| Cytochrome P450, family 24, subfamily A, polypeptide 1 | CYP24A1 | 0.3 | Oxidation Reduction |
| **NT5E** 5'-nucleotidase, ecto (CD73) | NT5E | 0.3 | Migration and invasion |
| Mitogen-activated protein kinase kinase kinase kinase 4 | MAP4K4 | 0.4 | Activate MAPK8/JNK |
| Caveolin 1, caveolae protein, | CAV1 | 0.4 | cell cycle progression and tumor suppressor |
| Poliovirus receptor-related 3 | PVRL3 | 0.4 | Cell adhesion |
| Hyaluronan-mediated motility receptor (RHAMM) | HMMR | 0.4 | tumor suppressor |
| Nuclear receptor subfamily 2, group F, member 1 | NR2F1 | 0.5 | TNFalpha signaling pathways |
| Ets variant 4 | ETV4 | 0.6 | Motility and invasion |
