# Supplementary figures and images for: Activated KrasG12D is associated with invasion and metastasis of pancreatic cancer cells through inhibition of E-cadherin
Source: Br J Cancer. 2011 Mar 1;104(6):1038–48. doi: 10.1038/bjc.2011.31 (PMC3065271; doi:10.1038/bjc.2011.31)

## Slide 1
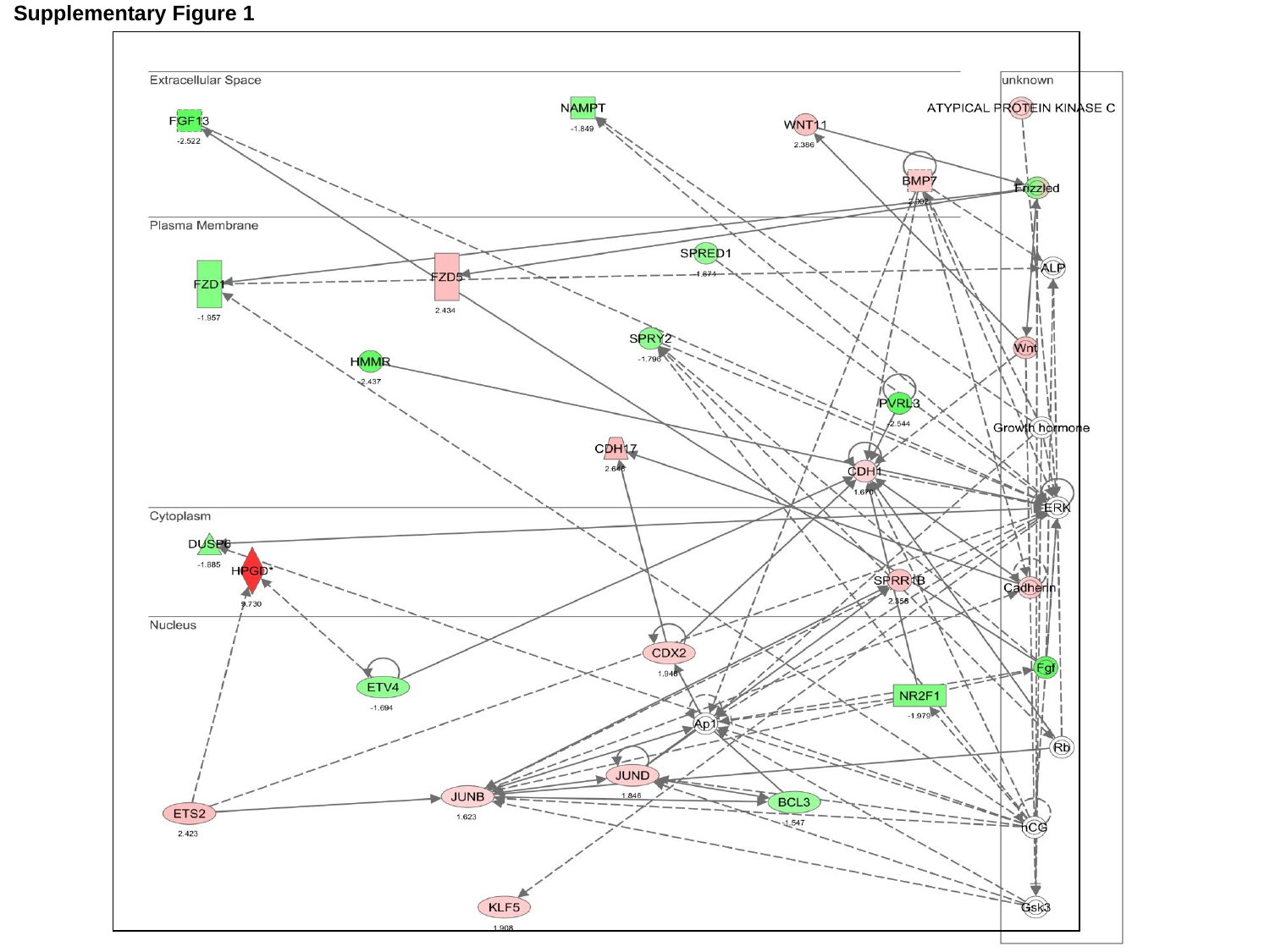

Supplementary Figure 1

Supplement: Supplementary Figure 1 [file bjc201131x4.ppt]
