# Supplementary material for: Activated KrasG12D is associated with invasion and metastasis of pancreatic cancer cells through inhibition of E-cadherin
Source: Br J Cancer. 2011 Mar 1;104(6):1038–48. doi: 10.1038/bjc.2011.31 (PMC3065271; doi:10.1038/bjc.2011.31)
Supplement: Supplementary Figure 1 Legend [file bjc201131x5.doc]

**Supplementary figure 1:** Gene networks identified based on the pathway prediction analysis. The genetic networks (HPGD, ALDH1A1, CDH1, and ETV4) were identified to be highly perturbed in response to downregulation of Kras^G12D^. Each node shape depicts the function of the gene product and the edge type shows the type of interaction between the molecules in the network. Nodes in grey are the actual genes found to be dysregulated in microarray analysis, while nodes in white are the genes taken from the dataset of the web application (Ingenuity pathways) knowledge base to generate gene-networks. The present analysis is indicative of the cross-talk of Kras signaling with other molecular pathways in pancreatic adenocarcinoma.
